# Assessing the Health of LiFePO_4_ Traction Batteries through Monotonic Echo State Networks

**DOI:** 10.3390/s18010009

**Published:** 2017-12-21

**Authors:** Luciano Sánchez, David Anseán, José Otero, Inés Couso

**Affiliations:** 1 Computer Science Department, Universidad de Oviedo, 33203 Gijón, Spain; jotero@uniovi.es; 2 Electrical Engineering Department, Universidad de Oviedo, 33203 Gijón, Spain; anseandavid@uniovi.es; 3 Statistics Department, Universidad de Oviedo, 33203 Gijón, Spain; couso@uniovi.es

**Keywords:** soft sensor, battery model, monotonic model, echo state networks

## Abstract

A soft sensor is presented that approximates certain health parameters of automotive rechargeable batteries from on-vehicle measurements of current and voltage. The sensor is based on a model of the open circuit voltage curve. This last model is implemented through monotonic neural networks and estimate over-potentials arising from the evolution in time of the Lithium concentration in the electrodes of the battery. The proposed soft sensor is able to exploit the information contained in operational records of the vehicle better than the alternatives, this being particularly true when the charge or discharge currents are between moderate and high. The accuracy of the neural model has been compared to different alternatives, including data-driven statistical models, first principle-based models, fuzzy observers and other recurrent neural networks with different topologies. It is concluded that monotonic echo state networks can outperform well established first-principle models. The algorithms have been validated with automotive Li-FePO_4_ cells.

## 1. Introduction

Battery costs comprise an estimated 25–50% of the electric vehicle [1]. Maximizing the life expectancy of a battery has undeniable economic implications, hence preventing the conditions that shorten Lithium battery life (high temperatures, overcharging, deep discharges, high current, etc.) is a major concern. Despite this, to the best of our knowledge, there are not reliable sensors that can measure the State of Health (SoH) of batteries that are already installed in a vehicle. Most of the SoH estimators are laboratory tools that cannot be easily adapted for on-vehicle condition monitoring.

Laboratory techniques for determining the SoH comprise direct measurements and electrochemical models [2]. There are advantages and drawbacks for the two families of methods. On the one hand, direct measurements can provide a view of the current state of the battery, but this view cannot be extrapolated to the future, i.e., direct measurements cannot always anticipate an incipient deterioration. On the other hand, electrochemical models have predictive capabilities, but these depend on many physical parameters of the battery. The evolution in time of these physical parameters is uncertain, hence electrochemical models do not perform well in mutable scenarios. Moreover, laboratory techniques for diagnosing a deterioration require, in certain cases, destructive operations (measuring the capacities of the positive and negative electrodes, the loss of Lithium inventory, etc.).

To a certain extent, these problems can be addressed through computer simulation. If a sufficiently precise battery model is available, it can be used for “virtual laboratory” experiments, where those off-vehicle laboratory measurements mentioned before are applied to a computer simulation of the battery. Machine learning techniques can be used to keep this model in sync with the current state of the battery. These learning algorithms operate with sequences of voltages and currents streamed by on-vehicle sensors [3]. The combination of a learning battery model and a suitable virtual lab procedure, with the purpose of synthesizing health-related variables from operational data, will be referred to as a “soft sensor” in this contribution [4].

The physical properties of a battery evolve with time, thus a learning battery model is needed. Many different battery models have been studied (a bibliographic study will be carried on in Section 2). In this paper, a soft sensor is proposed that combines transformation models [5] with reservoir computing [6] in a new class of monotonic Echo State Networks (ESN). Monotonic ESNs will serve as a dynamical model of the over-potentials arising from the nonlinear profiles of the Lithium concentration in the electrodes of the battery [7]. The new soft sensor is able to exploit the health-related information contained in operational records of the vehicle better than the alternatives, particularly when the charge or discharge currents are moderate to high. To validate this assertion, the accuracy of the new sensor has been compared, over automotive Li-FePO${}_{4}$ cells, to a selection of model-based observers of the state of health, including data-driven statistical models, first principle-based models, fuzzy observers and recurrent neural networks with different topologies.

This paper is organized as follows: Section 2 contains a brief bibliographic study about battery SoH assessment. In Section 3, the proposed Monotonic Echo Serial Network is introduced. An empirical study is introduced in Section 4, where the newly proposed sensor is compared to relevant alternatives. The paper concludes in Section 5.

## 2. State of the Art: Machine Learning Methods Suitable for Battery SoH Assessment

The functional dependence between the stored charge and the Open Circuit Voltage (OCV) of the battery at equilibrium contains information related to the most common deteriorations of a battery, such as the losses of Lithium inventory, active masses of anode and cathode [8,9]. Many different techniques exist for determining the OCV of a battery from data: “black boxes” or pure data-driven techniques such as statistical models, time series [10] and neural networks [11]; “grey boxes”, such as equivalent circuits [12] or fuzzy semi-physical models [13] and “white boxes” or first-principle models, that are based on the knowledge about the electrical and chemical processes that occur while the battery is being operated.

Soft sensors can be regarded as software-based observers of the state variables of a dynamical system. Examples of technologies suitable for its use in soft sensors are ARIMAX or NARMAX time series (Auto Regressive Integrated Moving Average with Explanatory Variable, and Nonlinear AutoRegressive Moving Average with eXogenous input, respectively) [14], Recurrent Neural Networks [15] or NARX (Nonlinear AutoRegressive with eXogenous input) neural or fuzzy models [16]. Fuzzy rule-based models allow incorportating expert knowledge in the form of “if-then” rules, and can take different forms: for example, there are fuzzy models of the nonlinearities in weakly linear models [17], state-space models with fuzzy rule-based parameters [18], and Wiener or Hammerstein Fuzzy Systems, where a linear dynamical system is followed by a static nonlinearity [19,20]. Recurrent Neuro-Fuzzy models [21] and NARX Neuro-Fuzzy Systems [22] have also been used in the past for modeling dynamic systems. For instance, ANFIS [23] has been arranged in a NARX configuration in dynamical problems [23].

Not all preceding methods can be used for assessing the health of a battery. NARX models are not appropriate for systems with a strong dependence on the initial conditions [24,25], but other recurrent neural networks are suitable for this problem. For example, Simple Recurrent Netwoks (SRNs), such as Elman or Jordan models [15], that are learnt with Backpropagation Through Time (BTT), can model these systems. The combination of SRNs and BTT is prone to the “vanishing gradient” problem [26], hence SRNs may not the best option; other neural architectures are more efficient than SRNs for events with a long time lag, as happens with batteries. Among these new architectures, Long Short-Term Memory (LSTM) and Reservoir Computing (RC) networks exhibit promising properties. LSTMs are based on units composed of memory blocks with gates that control the flow of information through the cell, where the weights control the operation of the gates [27,28]. Alternatively, Reservoir Computing (RC) is another recent extension that relies on a fixed, complex dynamical system (the “reservoir”) that is mapped to the desired output by means of a trainable readout mechanism. In particular, in Echo State Networks, the reservoir is a large recurrent neural network with randomly assigned weights, and the readout is a single neural layer whose weights are updated with ridge regression [29,30]. There are some variants of the ESN where the readout is nonlinear, such as φ-ESNs [31], or there is an additional feed-forward net between the inputs and the outputs that models short lags, such as ESN-R2SP (Echo State Network - Reservoir with Random Static Projections) [32]. These configurations can be benefitial when modelling highly non-linear systems, and its potential for diagnosing batteries has not been explored yet.

In this paper, a nonlinear readout ESN is used, but the learning process is different from that of φ-ESNs. The weights of the nonlinear readout are not optimized to reduce the squared error between the output of the net and the desired output, but an intermediate target function is sought that is comonotonic with the desired output of the net, and the nonlinear part is obtained via isotonic regression between intermediate and output variables, as described in the following section.

## 3. System Identification through Prediction Error and Transformation Models

Most machine learning techniques for identifying nonlinear systems entail the use of Prediction Error Methods (PEMs). PEMs are based on a parametric definition of the model, and the learning task consists in finding the set of parameters minimizing the prediction error for a given sequence of inputs and outputs [33]. As mentioned, in grey and white boxes, the knowledge that is discovered in the data is combined with different amounts of prior knowledge about the problem domain. This domain knowledge can be explicit, taking the form of constraints on the values of the learned parameters, or be implicitly built in the structure of the model, as done for instance with semi-physical models [34].

Embodying domain knowledge in the learning improves the model when the data is insufficient, but it can introduce additional “systematic” errors. This is so because “first principle” models depend on assumptions that do not always hold in practice. A balance must exist between those elements of the dynamic behavior of the system that are taken for granted and those that are learnt from data. It is often accepted, as a basic principle, that the amount of prior knowledge in a data-driven model has to be kept as low as possible.

A recent approach for encoding a small amount of prior knowledge in a model consists of enforcing the monotonicity of certain nonlinear blocks in the transference function. For instance, any digitally sampled system can be regarded as the composition of a continuous system and a staircase function. Saturations, dead zones, backlashes and different kinds of hysteresis also match this kind of “monotonic” prior information. The best studied cases are arguably monotonic Hammerstein or Wiener models, that consist of a composition of a linear system with a nonlinear monotonic function [35].

### Transformation Models: A Proposed Monotonic Echo State Network

Monotonic dynamical models can be learnt through transformation models [5]. Formally, let (f,θ) be a dynamical model defined by a parameter θ, applied to an input variable ${\left\{u_{t}\right\}}_{t}$, $u_{t}\in R^{m}$, and a nonlinear function f:R→R applied to the output of the dynamical model. Let also the output of the dynamical model be ${\left\{z_{t}\right\}}_{t}$, $z_{t}\in R$. The output of (f,θ) is the sequence ${\left\{{\hat{y}}_{t}\right\}}_{t}$, where ${\hat{y}}_{t}\left(f,\theta \right)=f\left(z_{t}\right)$. This sequence depends on the input sequence ${\left\{u_{t}\right\}}_{t}$, the parameter θ and the nonlinear function *f*.

Given a pair of sequences ${\left\{y_{t}\right\}}_{t}$ (desired output) and ${\left\{u_{t}\right\}}_{t}$ (observed input), the purpose of the learning algorithm is to find the value of θ and the function *f* for which the sequence ${\left\{{\hat{y}}_{t}\right\}}_{t}$ (model output) best approximates the desired output of the system ${\left\{y_{t}\right\}}_{t}$. PEMs aim to minimize the following error:

(1) $$\mathrm{error}\left(f,\theta \right)=\sum_{t=d}^{T}{\left({\hat{y}}_{t}\left(f,\theta \right)-y_{t}\right)}^{2}.$$

On the contrary, transformation models do not minimize the prediction error but search for the simplest model whose output is comonotonical with the desired output of the system. For example, the MINLIP algorithm for Monotone Wiener Systems [36] aims to solve the following optimization problem:

(2) $$\begin{array}{c} \min\;\mathrm{complexity}\;(f)\\ \;s.t.\;{\hat{y}}_{t}\left(f,\theta \right)=y_{t},\;\mathrm{for}\;\mathrm{all}\;t=d+1,\ldots ,T. \end{array}$$

*d* is a time delay, used for ignoring the effect of the initial conditions (state of the system at t=0). In the presence of noise, the transformation model is formed by introducing residuals $e_{t}$ such that ${\hat{y}}_{t}\left(f,\theta \right)=f\left(z_{t}+e_{t}\right)$. The learning becomes:

(3) $$\begin{array}{c} \min\mathrm{complexity}\left(f\right)\;\mathrm{and}\;|e_{t}|\\ \;s.t.\;f\left(z_{t}\left(\theta \right)+e_{t}\right)=y_{t},\;\mathrm{for}\;\mathrm{all}\;t=d+1,\ldots ,T. \end{array}$$

The nonlinear function *f* is defined by means of a isotonic regression algorithm on the set of pairs ${\left\{\left(z_{t},y_{t}\right)\right\}}_{t}$ [5]. Finally, the complexity of *f* is measured through its Lipschitz constant, which is the lowest value *L* such that

(4) $$|f\left(z_{i}\right)-f\left(z_{j}\right)\left|\leq L\right|z_{i}-z_{j}|\;\mathrm{for}\;\mathrm{all}\;i,j.$$

In order to define the proposed Monotonic Echo State Networks, let us rewrite first Equation (3) as follows:

(5) $$\begin{array}{c} \min\mathrm{complexity}\left(f\right)\;\mathrm{and}\;|e_{t}|\\ \;s.t.\;\mathrm{the}\;\mathrm{sequences}\;\;{\left\{z_{t}\left(\theta \right)+e_{t}\right\}}_{t=d+1,\ldots ,T}\;\mathrm{and}\;{\left\{y_{t}\right\}}_{t=d+1,\ldots ,T},\;\;\mathrm{are}\;\mathrm{comonotonic}. \end{array}$$

**Figure 1:**
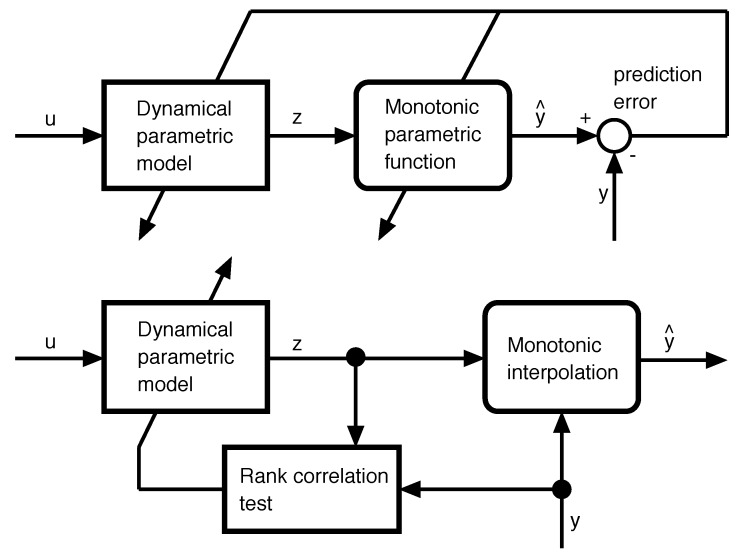
Prediction Error Models (PEMs) vs. the proposed formulation of transformation models: Upper part: PEMs find the parametric expressions of the dynamic model and the monotonic function by minimizing the prediction error of the model. Lower part: The purpose of transformation models is to learn a dynamical model whose output is comonotonic with the desired output. The monotonic function is not given a parametric expression but is obtained by interpolation (in the noiseless case) or isotonic regression (noisy data).

By means of Equation (5), the minimization of $|e_{t}|$ can be combined with the comonotonicity constraint by means of a rank correlation test τ [37], giving the following unconstrained optimization problem (see Figure 1):

(6) $$\begin{array}{c} \min\mathrm{complexity}(f)\\ \max\tau ({\left\{z_{t}\left(\theta \right)\right\}}_{t=d+1,\ldots ,T};{\left\{y_{t}\right\}}_{t=d+1,\ldots ,T}). \end{array}$$

Observe that the values $e_{t}$ do not appear anymore in Equation (6), as their effect is subsumed in the rank correlation test.

**Figure 2:**
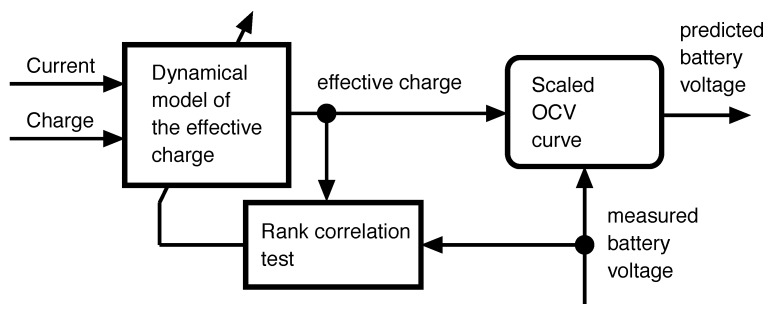
Transformation model of a battery: the perceived voltage of the battery when it is being charged (or discharged) matches the Open Circuit Voltage (OCV) at an instrumental cell at equilibrium, with a charge that is higher (or lower) than the actual charge. Since the OCV is monotonically increasing with respect to the charge, the instrumental or “effective” charge is also comonotonical with respect to the measured voltage.

The particularization of this structure to battery models follows. According to [7], the perceived voltage of an Li-Ion rechargeable battery depends on the Nernstian equilibrium potentials of the different phases of the lithiated graphite, Li${}_{x}$C${}_{6}$. The concentrations of the different phases depend, in turn, on the charge current through a diffusion process, with complex dynamics. During charge, the voltage of the battery is higher than the equilibrium voltage because the concentrations of the different stages of Li${}_{x}$C${}_{6}$ have not yet reached their final equilibrium. As a result, the voltage of the battery is the same as the voltage of an (hypothetical) instrumental cell at equilibrium, whose charge is higher than that of the actual battery. When the charge current is extinguished, the charge of the instrumental cell slowly converges to that of the actual cell. The opposite happens during discharge, when the instrumental battery has a lower charge than the actual battery. Let us call “effective charge” to the charge of the instrumental battery (see Figure 2). Observe that the effective charge and the actual battery voltage are comonotonical because they are related through the (scaled) OCV curve of the battery, which is monotonical by definition.

**Figure 3:**
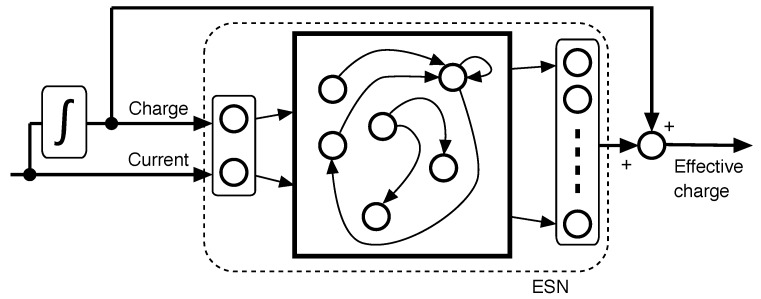
Dynamical model of the effective charge: the dynamical model of the effective charge comprises an Echo State Network (ESN) with two inputs: current and charge (integral of the current). The output of the ESN is the difference between the actual charge and the effective charge.

The desired outcome of the learning process is the scaled OCV curve, which can be exploited in turn to obtain the health information about the battery [38]. Given that the expression of the dynamical model of the effective charge is not needed, any black-box model is adequate. In this study, an Echo State Network [29] with linear activation layer is used, as shown in Figure 3. The block composition in Figure 2, in the particular case that the dynamical model is an ESN, will be referred to as “Monotone Echo State Network”.

The inputs to the ESN are the current and the charge (its integral), and the output is the difference between the actual charge of the battery and the effective charge defined before. Note, however, that the desired output of the neural network is unknown in this problem, as we only know that it is comonotonical with the perceived voltage. The weights of the output layer cannot be determined by ridge regression [30], but a gradient descent algorithm is used for minimizing a rank correlation test between the output of the net and the voltage of the battery.

## 4. Empirical Study and Discussion

Experiments in this section are designed for obtaining different battery health parameters through model-based OCV curve approximations. This experimentation serves two purposes: (a) to find out whether the proposed data-driven method is competitive with state-of-the-art dedicated models and (b) to verify that the model is accurate enough for gaining insight into the SoH of the battery.

**Figure 4:**
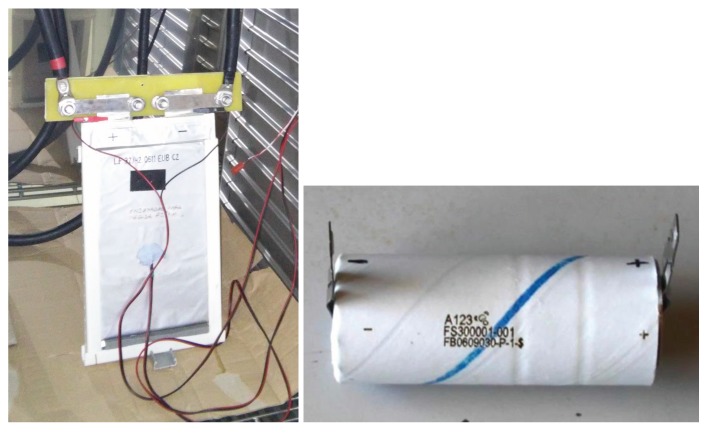
LiFePO${}_{4}$ (LFP) pouch battery from European Batteries (Varkaus, Finland) (**left**) and cell from manufacturer A123 Systems (Livonia, MI, USA) (**right**).

The experiments were conducted on three different batteries: Battery #1 is a 42Ah pouch battery from European Batteries, and batteries #2 and #3 are cylindrical commercial Lithium Iron Phosphate (LFP) cells manufactured by A123 Systems (Livonia, MI, USA), with 2.3 Ah name plate capacity (see Figure 4). These batteries are selected so that the dependence on the following conditions can be analyzed:

1. *Influence of the charge/discharge rate in the accuracy of the sensor.* Battery #1 was used for this purpose, and was charged at 42, 21, 14, 8.4 and 1.68 Amps (these currents are named C1, C2, C3, C5 and C25). It is expected that the soft sensor is effective for C25 and also that its quality degrades for the higher currents.
2. *Influence of the ageing of the battery.* Batteries #2 and #3 were subjected to 6000 charge/discharge cycles and different experiments were programmed at the beginning of their lifes, at half life (3000 cycles) and at the end of their useful life (6000 cycles). Battery #3 had an abnormal deterioration (electrodeposition). Battery #2 had a normal ageing with a gradual reduction of the capacity until the end of its life.
3. *Influence of the technology.* Battery #1 is a pouch battery; #2 and #3 are cylindrical, and the capacities are also different; Battery #1 is a large cell (42 Ah, used, for instance, in battery-electric buses) and the other cells are much smaller (2.3 Ah, used for instance in the BMW ActiveHybrid 3 Hybrid Electric Vehicles HEVs (Munich, Germany) or the Chevrolet Spark EV (Detroit, MI, USA)).

The LiFePO${}_{4}$ (LFP) pouch battery from European Batteries (Varkaus, Finland) (see Figure 4) has a rated capacity of 42 Ah when discharged at 8.4 Amps. The average operating voltage is 3.2 V. The discharge and charge cut-off voltages are 2.5 V and 3.65 V, respectively. The dimensions in mm are 275 × 166.5 × 13.3. The cell weighs 1010 g. The cylindrical battery from A123 Systems has a rated capacity of 2.3 Ah when discharged at 2.3 Amps. The average operating voltage is 3.3 V. The discharge and charge cut-off voltages are 2 V and 3.6 V, respectively. The dimensions in mm are ∅ 26 × 65. The cell weighs 76 g.

**Figure 5:**
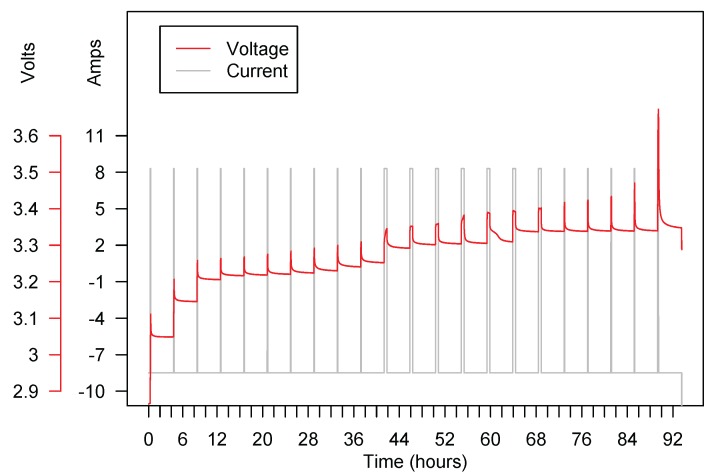
Relaxation experiment for obtaining the OCV curve of the 42 Ah battery. The battery is charged in steps of 5% of the capacity and left resting for some hours before the next charge step is applied. Each of these steps produces a pair (voltage, capacity) of the OCV curve.

Tests are conducted in an SBT10050 battery test system from PEC (Leuven, Beligium) and an ICP750 climate chamber from Memmert (Schwabach, Germany). The ambient temperature was 23 °C. The OCV of the 42 Ah battery has been measured through the “voltage relaxation” method [39]. This method consists of charging the battery at constant current in small steps (about 5–10% of the capacity) and then applying a constant voltage until the current is smaller than a threshold to ensure a full charge (or discharge). Each of these steps is followed by a rest period of some hours, after which the OCV voltage is measured (see Figure 5 for the actual current and voltage profiles obtained in the relaxation experiment.) The OCV of the smaller A123 batteries has been obtained as the average, for a set of stored charges, of the voltages of the C25 charge and discharge curves.

### 4.1. Assessment of the Neural Model

The first part of the experimentation consists of a numerical comparison of the accuracies of the present approach and other OCV models in terms of the residual of the approximation of the model to the “ground truth” OCV measured at the laboratory. The amplitude of the residual is measured through the following expression:

(7) $$\mathrm{error}=\frac{1}{N}\sum_{i=1}^{N}{\left(OCV_{\mathrm{true}}\left(q_{i}\right)-OCV_{\mathrm{model}}\left(q_{i}\right)\right)}^{2},$$

where $q_{1}\ldots q_{n}$ are the charges at the different resting phases, and *N* is the number of phases.

**Figure 6:**
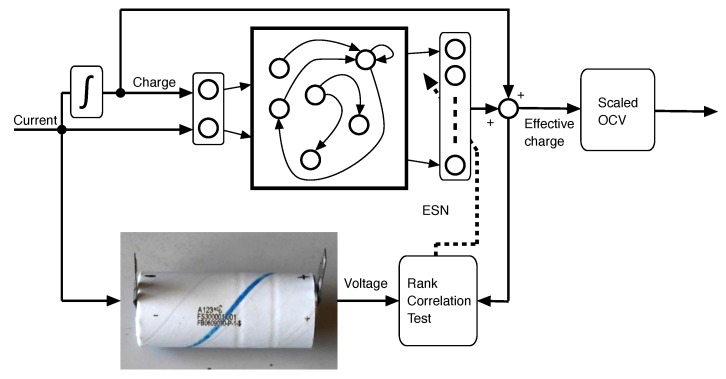
Virtual laboratory-based training: training data is sampled while the battery is being used. The rank correlation test between the output of the Monotone Echo State Network (MESN) (effective charge) and the actual voltage of the battery is maximized. The OCV is obtained by applying isotonic regression between the output of the trained MESN and the actual voltage.

**Figure 7:**
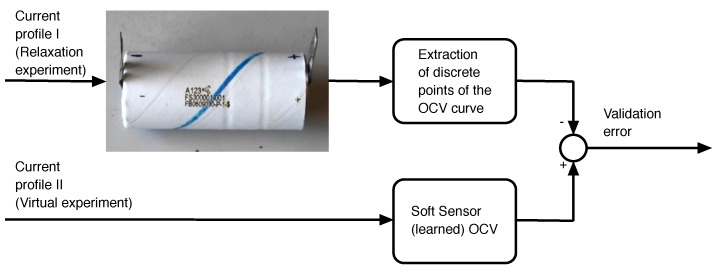
Virtual laboratory-based validation: the OCV curve obtained in the training is compared to a discrete set of points of the actual OCV curve of the battery, obtained in an independent relaxation experiment, carried out in controlled conditions of load and temperature.

It is remarked that the experimental design of this study is unlike the standard practice in machine learning experiments. In this problem, train and test datasets are not obtained with cross validation, but the battery model is subjected to a “virtual lab” experiment. With the expression “virtual lab”, we mean that a battery model is learned from on-vehicle data first. This model is subsequently used to simulate a full charge at a very low current (a virtual experiment). The estimation of the (pseudo) OCV curve is the set of pairs (charge, voltage) predicted by the learnt model during this simulated experiment (see Figure 6 and Figure 7).

The following eight computer models are included in this study. The residuals of the approximation of these models (see Equation (7)) and compared to that of the proposed MESN model:

1. Abu–Sharkh’s method [40]. State-of-the-art method for determining the OCV of a battery from operational data, based on a first-principles model of the battery.
2. Xu’s method [41]. Another specialized method that is based on Randles’ equivalent circuit.
3. LSTM [26]. An LSTM recurrent neural network with 20 hidden nodes with recurrent connections followed by a linear layer. The net is primed with two sequences of inputs: the current and the charge.
4. LSTM-dropout [28]. A regularized LSTM network where randomly chosen network units are masked during training.
5. ESN [42]. Echo State Network, with a reservoir of size 1000, and a linear feed-forward layer trained with ridge regression.
6. φ-ESN [31] An ESN with an additional nonlinear feed-forward layer, where the non-recurrent layers are trained with the Adam algorithm [43].
7. ANFIS (Adaptive-Network-Based Fuzzy Inference System) [23] in a NARX configuration. ANFIS is a mature technique where a fuzzy system is designed to be functionally equivalent to a feed-forward net. Since NARX models are not well suited for this problem, this algorithm is included as a “worst case” metric.
8. ARIMAX(2,1): Auto Regressive Integrated Moving Average with Explanatory Variable time series, with orders AR = 2, MA = 1—also intended as a baseline.

The results of the study are displayed in Table 1, Table 2 and Table 3. The influence of the charge/discharge rate in the accuracy of the sensor is studied in Table 1. Observe that the accuracy of all methods degrades for high currents. Remarkably, neural networks were able to improve over battery specific algorithms, and MESN was able to keep the C25 accuracy for C5 discharges. In addition to this, the performance of MESN is better by an order of magnitude than any of the other methods for currents higher than C5 (i.e., C3, C2, C1). This is the main result of this contribution, as the purpose of this method is to measure the health of a battery from data sampled while the vehicle is in use, and the nominal current of this battery is higher than C5.

Table 2 measures the accuracy of the model when the batteries are at the beginning, middle and end of life and the ageing process has been uniform (without abnormal deterioration). In all cases, the charging current is C25. In these experiments, Abu–Sharkh and Xu’s methods were only evaluated in their most favourable configuration, i.e., for charges lower than 80% of the capacity of the battery. The accuracy of MESN is comparable or better than that of the specific methods, showing that the method is not negatively influenced by the battery age, as expected (because MESN does not depend on an electrochemical model of the battery; the only assumption related to the electrochemical properties of the battery was the comonotonicity of the charge and the voltage when the cell is at equilibrium).

**Figure 8:**
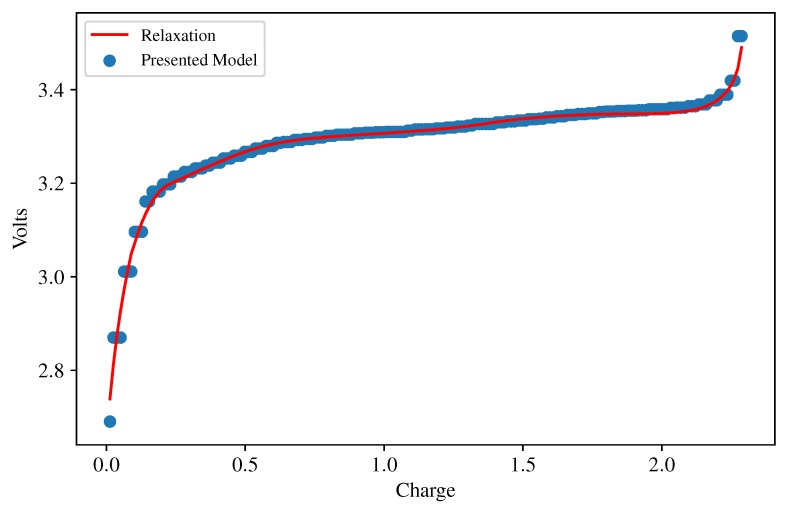
Model (blue dots) and ground truth (spline-interpolated points of the relaxation-obtained OCV curve) for the first A123 battery.

Lastly, Table 3 measures the accuracy of the model at the beginning, middle and end of life when an abnormal deterioration takes place (an electrodeposition happened at some point between the middle and the end of the life). There are not appreciable differences between the accuracy of the battery model in this case and the results in Table 2. Observe that MESN was not better than Abu–Sharkh’s method for the battery at the beginning of its life. In any case, the differences between these two methods are not significant, owing to the fact that MESN was the best method in the first column of Table 2, with another A123 cell with the same SoH. It is also remarked that MESN was able to obtain very good results for the degraded cells. In Figure 8, a graphical assessment of the accuracy of the OCV model is shown: the residual is low in the flat area of the curve, and moderate in the areas with strong slopes (battery charge lower than 10% or higher than 90%).

### 4.2. Extraction of Health Parameters from the OCV Model

**Figure 9:**
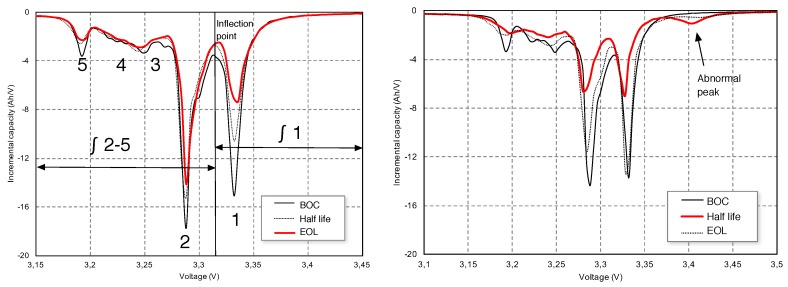
Pseudo-OCV Incremental Capacity Analysis (ICA) from a controlled C25 discharge. The labelling of the peaks of the curve is taken from [38]. (**left**) name convention and ICA curves for battery A123 without electrodeposition and the beginning, half life and end of life; (**right**) battery A123 with electrodeposition.

The second part of the experimentation is guided to obtain SOH parameters from the OCV model and to compare them to estimations of the same parameters that are taken from the “ground truth” OCV curve. Incremental capacity Analysis (ICA) of the equilibrium open-circuit voltage is used to find the signatures of the different deteriotations in the OCV [44]. It is remarked that standard ICA analysis are not obtained from OCV estimations, as proposed in this paper, but from controlled discharges at C25 at the laboratory (see Figure 9). There are five characteristic points in these curves [38] that can be related to different degradations.

**Figure 10:**
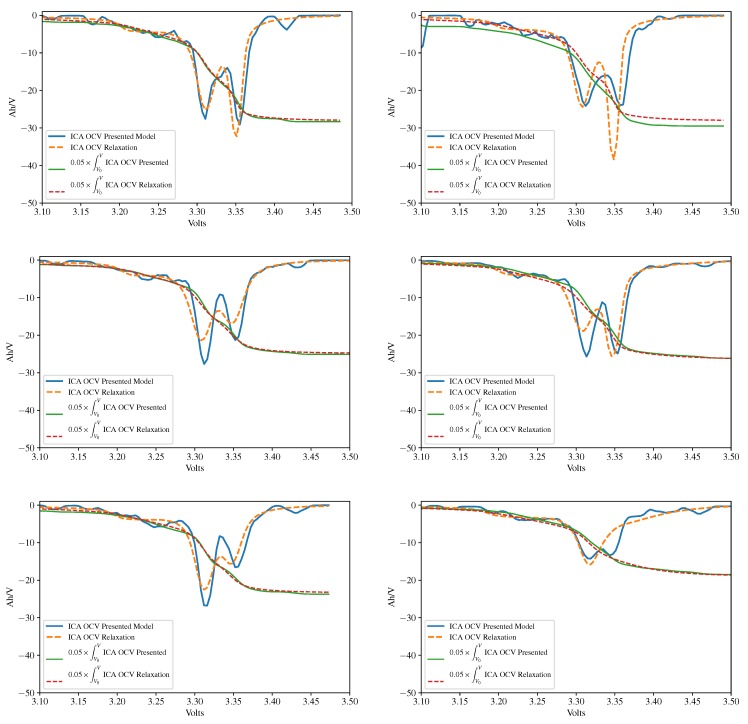
Pseudo-OCV analysis for OCV curves estimated at the laboratory (dashed lines) and on-vehicle with the present model (continuous lines). **Left column**, from upper to lower: battery A123 without electrodeposition. **Right column**: battery A123 with electrodeposition. The ICA curves (derivative of the OCV with respect to stored charge) and the integral of these curves are plotted in the same graphs. The accuracy of the proposed method is comparable to that of the relaxation experiment, as both the integral of the curves and the position of the inflection point are similar.

The outputs of the proposed soft sensor, for batteries #2 and #3 at the beginning of their cyclings, half and end of life are collected in Table 4 and Table 5. A graphical analysis of the same data is provided in Figure 10. Let us recall that the presented method obtains the OCV curve from on-vehicle data. It is not expected that on-vehicle estimations are as precise as laboratory measurements. The differences between the estimation and the ground truth are very small in the least squares sense (see Table 1, Table 2 and Table 3 and Figure 8) and indeed may seem negligible, but it is possible that a model converges to the true values in the least square sense, and, at the same time, the derivatives of this model do not converge to the true derivatives. To a certain extent, this problem occurs in the case being studied because the output of the neural net is discontinuous and therefore non-differentiable. A moderate smoothing had to be applied in order to obtain the ICA curves. This smoothing must be small enough for not distorting peaks 1 and 2. As a consequence of this, it is possible that some short peaks appear in the ICA curve that are artifacts of the numerical methods (see, for instance, the peaks on the right side—between the 3.40 and 3.45 Volt marks—of the blue curves in the left column of Figure 10).

Observe also in Figure 10 that the ICA curves obtained from the estimation have noticeable differences with respect to those obtained from the relaxation curves in Figure 9. The positions of the five peaks in this last figure cannot be recovered because model-estimated OCV curves have lower resolution than laboratory measurements. The derivatives of the voltage with respect to the charge were taken at steps of 40 mV. Hence, peaks 3, 4, and 5 cannot be reliably detected.

In any case, there is useful information about the battery health that is kept in the approximation. In particular, we show that the approximation is good enough for obtaining the location of the inflection point and also Peak areas 1 and 2–5. The evolution of these areas is enough for guessing whether the main deterioration cause is a Loss of Lithium Inventory (LLI) or a shrinking of the lithiathed graphite zone (LAM${}_{\mathrm{deNE}}$) (see Reference [45]). Hence, in this study, we will focus ourselves in the position of the inflection point between peaks 1 and 2 and the area of the ICA curve to the left and right of this inflection point. The evolution of these areas is enough for detecting “silent deteriorations”, i.e., those degradations that are not detected through capacity fade. The ICA curves (derivatives of the OCV with respect to stored charge) and the integrals of these curves are plotted in the same graphs (Figure 10), showing that the accuracy of the proposed method is comparable to another diagnosis that was based on the “ground truth” OCV curve, as both the integral of the curves and the position of the inflection point are similar.

For Battery #2 (see Table 4), we conclude that the diagnostic is the same with both relaxation and the presented on-vehicle method: Peak area 1 decreases, and Peak areas 2–5 are almost constant, thus the main deterioration cause is LLI. Observe that two values were marked with an asterisk in this last table. These values are regarded as inaccurate because they are not in complete agreement with the measurements taken on the ICA curve that are measured on the C25 discharge. They are a consequence of the use of the OCV curve for performing the diagnosis and not a drawback of the presented method.

The most remarkable result in this experiment analysis is obtained for Battery #3, where the presented method was able to predict an electrodeposition before it happened. Our method found that Peak 1 was growing; according to References [46,47], an early detection of Lithium plating can be used to reduce the duty-cycling scheme requirements, hence avoiding further degradation and prolonging cycle-life. Laboratory and on-vehicle methods produce the same result: Peak area 1 does not decrease, Peak areas 2–5 decrease, thus there is LLI and the effect of the shrinking of the lithiathed graphite zone (LAM${}_{\mathrm{deNE}}$) is significant, in agreement with the presence of the abnormal peak in the right part of Figure 9.

## 5. Conclusions

A new soft sensor for measuring certain health parameters of automotive batteries through analysis of its OCV curve has been presented. This sensor could be embedded in SoH assessment systems. The proposed sensor is based on a recurrent neural network that implements a transformation model of the battery. The results were validated for LiFePO${}_{4}$ batteries. Eight different OCV models were compared to the new approach: two first-principle models, five recurrent neural networks and statistical time series. It was concluded that (a) MESN networks improve state-of-the-art methods for OCV modeling when the charge current is moderate or high and (b) health diagnostics from OCV models are useful for obtaining on-vehicle assessments of the battery health, including certain silent deteriorations.

To the best of our knowledge, this is the first application of transformation models in battery modelling. The method uses only a small amount of prior knowledge, which is the monotonicity of the OCV with respect to the charge. Since this monotonicity assumption is valid for a large number of scenarios (different currents, ageing and degradations), this technique is less restrictive than electrochemical or equivalent circuit models. 

## Figures and Tables

**Table 1:** Influence of the charging current. Average quadratic error of Open Circuit Voltage (OCV), obtained from Recurrent Neural Networks (RNN), Long-Short Term Memory (LSTM), Echo State Networks (ESN), γ-Echo State Networks (γ-ESN), Adaptive Neuro-Fuzzy Inference Systems (ANFIS), Autoregressive Integrated Moving Average with Explanatory Variable (ARIMAX) and Monotone Echo State Networks (MESN).

	C25	C5	C3	C2	C1
Abu–Sharkh	**0.0003**	0.0094	0.0080	0.0084	0.0110
Xu	0.0006	0.0086	0.0146	0.0153	0.0073
LSTM	0.0077	0.0301	0.0070	0.0066	0.0064
LSTM-dropout	0.0100	0.0295	0.0067	0.0083	0.0093
ESN	0.0056	0.0326	0.0083	0.0106	0.0083
γ-ESN	0.1553	0.0854	0.0279	0.0132	0.0212
ANFIS	0.2026	0.2016	0.0731	0.0595	0.0334
ARIMAX(2,1)	0.0127	0.0391	1.0153	0.0165	0.0202
MESN	**0.0003**	**0.0003**	**0.0007**	**0.0007**	**0.0018**

**Table 2:** Influence of the number of cycles without electrodeposition. Average quadratic error of OCV, obtained from RNN, LSTM, ESN, γ-ESN, ANFIS, and ARIMAX.

	New Battery	Middle Life	End of Life
Abu–Sharkh	0.0003	0.0008	0.0009
Xu	0.0008	0.0016	0.0015
LSTM	0.0016	0.0027	0.0069
LSTM-dropout	0.0015	0.0035	0.0033
ESN	0.0060	0.0131	0.0125
γ-ESN	0.0132	0.0120	0.1972
ANFIS	0.0573	0.0913	0.0808
ARIMAX(2,1)	0.0494	0.0619	0.0603
MESN	**0.0002**	**0.0005**	**0.0008**

**Table 3:** Influence of the number of cycles with electrodeposition. Average quadratic error of OCV, obtained from RNN, LSTM, ESN, γ-ESN, ANFIS, and ARIMAX.

	New Battery	Middle Life	End of Life
Abu–Sharkh	**0.0002**	0.0008	0.0010
Xu	0.0007	0.0016	0.0018
LSTM	0.0026	0.0026	0.0031
LSTM-dropout	0.0022	0.0019	0.0029
ESN	0.0054	0.0212	0.0639
γ-ESN	0.0062	0.1506	0.0343
ANFIS	0.0639	0.1709	0.1549
ARIMAX(2,1)	0.0620	0.0447	0.0828
MESN	0.0004	**0.0001**	**0.0001**

**Table 4:** Peak area, battery without electrodeposition. The diagnostic is the same with both relaxation and the presented on-vehicle method: Peak area 1 decreases, Peak areas 2–5 are almost constant, thus the main deterioration cause is Loss of Lithium Inventory (LLI). In addition, the values marked with an asterisk are inaccurate because they are not in complete agreement with our laboratory measurements; these values should be slightly smaller.

	Method	New Battery	Middle Life	End of Life
Peak Area 1	Relaxation	210	175	150
Peak Areas 2–5	Relaxation	360	330	330(*)
Peak Area 1	Presented method	180	175	150
Peak Areas 2–5	Presented method	390	330	330(*)

**Table 5:** Peak area, battery with electrodeposition. The diagnostic with relaxation is incomplete because peak 1 cannot be detected from the OCV curve for the resolution used in this experiment (4 mV). In any case, laboratory and on-vehicle methods produce the same result: Peak area 1 does not decrease, Peak areas 2–5 decrease, thus not only LLI but shrinking of the lithiathed graphite zone (Loss of Active Material, LAM${}_{\mathrm{deNE}}$) effects are significant. This is in agreement with the presence of the abnormal in the right part of Figure 9 (note that this peak cannot be reliably measured in Figure 10; the small peak in the figures in the left part is an artifact of the smoothing algorithm).

	Method	New Battery	Middle Life	End of Life
Peak Area 1	Relaxation	220	220	–
Peak Areas 2–5	Relaxation	340	310	–
Peak Area 1	Presented method	170	200	120
Peak Areas 2–5	Presented method	430	330	250

## References

[1] Nykvist B., Nilsson M. (2015). Rapidly falling costs of battery packs for electric vehicles. Nat. Clim. Chang..

[2] Barre A., Deguilhem B., Grolleau S., Gerard M., Suard F., Riu D. (2013). A review on Lithium-ion battery ageing mechanisms and estimations for automotive applications. J. Power Sources.

[3] Kadlec P., Gabrys B., Strandt S. (2009). Data-driven soft sensors in the process industry. Comput. Chem. Eng..

[4] Sánchez L., Couso I., Otero J., Echevarría Y., Anseán D. (2017). A Model-Based Virtual Sensor for Condition Monitoring of Li-Ion Batteries in Cyber-Physical Vehicle Systems. J. Sens..

[5] Belle V.V., Pelckmans K., Suykens J.A.K., Huffel S.V. (2011). Learning Transformation Models for Ranking and Survival Analysis. J. Mach. Learn. Res..

[6] Lukoševičius M., Jaeger H. (2009). Reservoir computing approaches to recurrent neural network training. Comput. Sci. Rev..

[7] Gallagher K.G., Dees D.W., Jansen A.N., Abraham D.P., Kang S.H. (2012). A volume averaged approach to the numerical modeling of phase-transition intercalation electrodes presented for LixC6. J. Electrochem. Soc..

[8] Birkl C.R., Roberts M.R., McTurk E., Bruce P.G., Howey D.A. (2017). Degradation diagnostics for Lithium ion cells. J. Power Sources.

[9] Weng C., Sun J., Peng H. (2014). A unified open-circuit-voltage model of Lithium-ion batteries for state-of-charge estimation and state-of-health monitoring. J. Power Sources.

[10] Saha B., Goebel K., Christophersen J. (2009). Comparison of prognostic algorithms for estimating remaining useful life of batteries. Trans. Inst. Meas. Control.

[11] Lu L., Han X., Li J., Hua J., Ouyang M. (2013). A review on the key issues for Lithium-ion battery management in electric vehicles. J. Power Sources.

[12] Seaman A., Dao T.S., McPhee J. (2014). A survey of mathematics-based equivalent-circuit and electrochemical battery models for hybrid and electric vehicle simulation. J. Power Sources.

[13] Sánchez L., Couso I., González M. (2014). A design methodology for semi-physical fuzzy models applied to the dynamic characterization of LiFePO_4_ batteries. Appl. Soft Comput..

[14] Ljung L. (1998). System Identification. Signal Analysis and Prediction.

[15] Haykin S.S. (2009). Neural Networks and Learning Machines.

[16] Lin T., Horne B.G., Tino P., Giles C.L. (1996). Learning long-term dependencies in NARX recurrent neural networks. IEEE Trans. Neural Netw..

[17] Bartczuk Ł., Przybył A., Cpałka K. (2016). A new approach to nonlinear modelling of dynamic systems based on fuzzy rules. Int. J. Appl. Math. Comput. Sci..

[18] Massad E., Ortega N.R.S., de Barros L.C., Struchiner C.J. (2008). Classical Dynamical Systems with Fuzzy Rule-Based Parameters. Fuzzy Logic in Action: Applications in Epidemiology and Beyond.

[19] Abonyi J., Babuska R., Szeifert F., Nagy L. (2000). Identification and Control of Nonlinear Systems Using Fuzzy Hammerstein Models. Ind. Eng. Chem. Res..

[20] Sánchez L., Couso I., Blanco C. (2017). A class of Monotone Fuzzy rule-based Wiener systems with an application to Li-ion battery modelling. Eng. Appl. Artif. Intell..

[21] Zhang J., Morris A.J. (1999). Recurrent neuro-fuzzy networks for nonlinear process modeling. IEEE Trans. Neural Netw..

[22] Babuška R., Verbruggen H. (2003). Neuro-fuzzy methods for nonlinear system identification. Annu. Rev. Control.

[23] Jang J.S. (1993). ANFIS: Adaptive-network-based fuzzy inference system. IEEE Trans. Syst. Man Cybern..

[24] Sjoberg J., Zhang Q., Ljung L., Benveniste A., Delyon B., Glorennec P.Y., Hjalmarsson H., Juditsky A. (1995). Nonlinear black-box modeling in system identification: A unified overview. Automatica.

[25] Juang J.N. (1994). Applied System Identification.

[26] Hochreiter S. (1998). The vanishing gradient problem during learning recurrent neural nets and problem solutions. Int. J. Uncertain. Fuzziness Knowl. Based Syst..

[27] Hochreiter S., Schmidhuber J. (1997). Long short-term memory. Neural Comput..

[28] Gal Y., Ghahramani Z. (2016). A theoretically grounded application of dropout in recurrent neural networks. Advances in Neural Information Processing Systems.

[29] Verstraeten D., Schrauwen B., Haene M., Stroobandt D. (2007). An experimental unification of reservoir computing methods. Neural Netw..

[30] Lukoševičius M. (2012). A practical guide to applying echo state networks. Neural Networks: Tricks of the Trade.

[31] Gallicchio C., Micheli A. (2011). Architectural and markovian factors of echo state networks. Neural Netw..

[32] Butcher J., Verstraeten D., Schrauwen B., Day C., Haycock P. Extending reservoir computing with random static projections: A hybrid between extreme learning and RC. Proceedings of the 18th European Symposium on Artificial Neural Networks (ESANN 2010).

[33] Ljung L. (2002). Prediction error estimation methods. Circ. Syst. Signal Process..

[34] Lindskog P., Ljung L. (1995). Tools for semiphysical modelling. Int. J. Adapt. Control Signal Process..

[35] Schetzen M. (2010). Nonlinear System Modelling and Analysis from the Volterra and Wiener Perspective. Block-Oriented Nonlinear System Identification.

[36] Pelckmans K. (2011). MINLIP for the identification of monotone Wiener systems. Automatica.

[37] Noether G.E. (1981). Why Kendall Tau?. Teach. Stat..

[38] Dubarry M., Truchot C., Liaw B.Y. (2012). Synthesize battery degradation modes via a diagnostic and prognostic model. J. Power Sources.

[39] Pei L., Lu R., Zhu C. (2013). Relaxation model of the open-circuit voltage for state-of-charge estimation in Lithium-ion batteries. IET Electr. Syst. Transp..

[40] Abu–Sharkh S., Doerffel D. (2004). Rapid test and non-linear model characterisation of solid-state Lithium-ion batteries. J. Power Sources.

[41] Xu J., Cao B., Chen Z., Zou Z. (2014). An online state of charge estimation method with reduced prior battery testing information. Int. J. Electr. Power Energy Syst..

[42] Maass W., Natschläger T., Markram H. (2002). Real-Time Computing Without Stable States: A New Framework for Neural Computation Based on Perturbations. Neural Comput..

[43] Kingma D.P., Ba J. Adam—A Method for Stochastic Optimization. Proceedings of the The International Conference on Learning Representations (ICLR).

[44] Dubarry M., Berecibar M., Devie A., Anseán D., Omar N., Villarreal I. (2017). State of health battery estimator enabling degradation diagnosis: Model and algorithm description. J. Power Sources.

[45] Anseán D., González M., Blanco C., Viera J.C., Fernández Y., García V.M. Lithium-ion battery degradation indicators via incremental capacity analysis. Proceedings of the IEEE International Conference on Environment and Electrical Engineering and 2017 IEEE Industrial and Commercial Power Systems Europe (EEEIC/I&CPS Europe).

[46] Anseán D., Dubarry M., Devie A., Liaw B., García V., Viera J., González M. (2016). Fast charging technique for high power LiFePO 4 batteries: A mechanistic analysis of aging. J. Power Sources.

[47] Anseán D., Dubarry M., Devie A., Liaw B., García V., Viera J., González M. (2017). Operando Lithium plating quantification and early detection of a commercial LiFePO 4 cell cycled under dynamic driving schedule. J. Power Sources.

